# Complete genome sequencing of the luminescent bacterium, *Vibrio qinghaiensis sp. Q67* using PacBio technology

**DOI:** 10.1038/sdata.2017.205

**Published:** 2018-01-16

**Authors:** Liang Gong, Yu Wu, Qijie Jian, Chunxiao Yin, Taotao Li, Vijai Kumar Gupta, Xuewu Duan, Yueming Jiang

**Affiliations:** 1 Key Laboratory of Plant Resource Conservation and Sustainable Utilization/Guangdong Provincial Key Laboratory of Applied Botany, South China Botanical Garden, Chinese Academy of Sciences, Guangzhou 510650, China; 2 University of Chinese Academy of Sciences, Beijing 100039, China; 3 Long Ping Branch, Graduate School of Hunan University, Changsha 410125, China; 4 Department of Chemistry and Biotechnology, ERA Chair of Green Chemistry, Tallinn University of Technology, Tallinn 12618, Estonia

**Keywords:** Bacterial genetics, Environmental sciences, DNA sequencing, Genome

## Abstract

*Vibrio qinghaiensis* sp*.-Q67* (Vqin-Q67) is a freshwater luminescent bacterium that continuously emits blue-green light (485 nm). The bacterium has been widely used for detecting toxic contaminants. Here, we report the complete genome sequence of Vqin-Q67, obtained using third-generation PacBio sequencing technology. Continuous long reads were attained from three PacBio sequencing runs and reads >500 bp with a quality value of >0.75 were merged together into a single dataset. This resultant highly-contiguous *de novo* assembly has no genome gaps, and comprises two chromosomes with substantial genetic information, including protein-coding genes, non-coding RNA, transposon and gene islands. Our dataset can be useful as a comparative genome for evolution and speciation studies, as well as for the analysis of protein-coding gene families, the pathogenicity of different *Vibrio* species in fish, the evolution of non-coding RNA and transposon, and the regulation of gene expression in relation to the bioluminescence of Vqin-Q67.

## Background & Summary

Luminous bacteria are a group of bacteria that have the ability to produce light, and are very common in ocean environments. However, some species of luminous bacteria have been found in terrestrial environments. *Vibrio qinghaiensis sp.-Q67* (Vqin-Q67) is a luminous bacterium that was first isolated from Qinghai Lake, Qinghai Province, China^1^. It has proved to be very sensitive in detecting environmental and food pollutants such as phthalate esters^2^ and fusaric acid^3^. The light-emitting mechanism of luminous bacteria is associated with the presence of *lux* genes coding luciferases, which catalyse the oxidation of a reduced flavin mononucleotide (FMNH2) and a long-chain aliphatic (fatty) aldehyde (RCHO) to generate blue-green light in the presence of O_2_ (ref. 4). A previous study showed that the luminescence of *V. fischeri* relies on activation of a transcriptional protein, LuxR, which is associated with N-3-oxo-hexanoyl-HSL (3OC6-HSL) and/or N-octanoyl-HSL (C8-HSL) to form a complex binding at the promoter site within the *lux* operon to activate transcriptional regulation of these light production-associated genes^5^. *V. fischeri* has been isolated from ocean environments^6^, while Vqin-Q67 has been identified as the only known luminous bacterium belonging to the family *Vibrionaceae* found in a fresh water enivronment. To date, very little information is know about the genome, or the genes involved in the luminescence, of Vqin-Q67.

**Figure 1:**
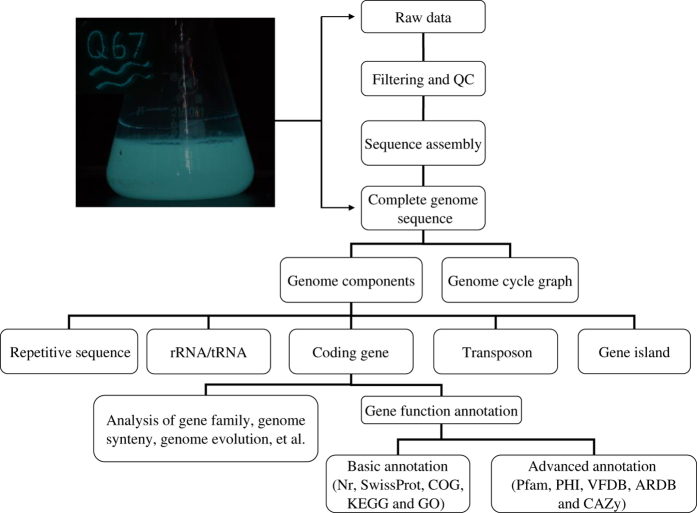
Overview of the experimental design of the study.

In the present study, Vqin-Q67 was cultured in modified Czapek’s broth medium containingNaHCO_3_ (1.345 g l^−1^), K_2_HPO_4_ (0.0136 g l^−1^), Na_2_HPO_4_ (0.0358 g l^−1^), MgCl_2_·6H_2_O (0.60 g l^−1^), CaCL_2_ (0.033 g l^−1^), MgSO_4_·7H_2_O (0.246 g l^−1^), NaCl (1.54 g l^−1^), yeast extract (5.0 g l^−1^), tryptone (5.0 g l^−1^) and glycerinum (3.0 g l^−1^)at 22 °C with shaking (120 rpm) for 48 h. When blue-green light became visible (Fig. 1), the bacterial cells were collected by centrifugation at 4,000×*g* for 10 min and then immediately stored in liquid nitrogen for further experimental analysis. The genome of Vqin-Q67 was sequenced as the flowchart shown in Fig. 1. The PacBio RS II system with P4/C2 chemistry (Pacific Biosciences, Menlo Park, CA, USA) recently has been developed a single-molecule real-time (SMRT) analysis^7^. The advantages of PacBio sequencing are the resulting highly-contiguous *de novo* assembly and longer read length (>10 Kb), which can close the gaps in assembled genome sequence and allow read-through of repetitive regions and. The aim of this study is to obtain a complete genome sequence for Vqin-Q67. This dataset reported here will be useful for analysis of protein-coding gene families, for comparative genomic analysis of evolution and pathogenicity among different *Vibrio* species, for analysis of non-coding RNA and transposon evolution at a whole genome level, and for analysis of the regulation of gene expression in relation to the bioluminescence of Vqin-Q67.

## Methods

### Genomic DNA preparation

Genomic DNA was extracted using E.Z.N.A. Fungal DNA Kit (Omega Bio-tek, Hangzhou, China) according to the manufacturer’s instructions. The DNA quality was evaluated using a Qubit fluorometer (Thermo Fisher Scientific, Waltham, MA) and Nanodrop spectrophotometer (Thermo Fisher Scientific).

### Sequencing

Qualified genomic DNA was fragmented using G-tubes (Covaris) and then end-repaired to prepare SMRTbell DNA template libraries (with a fragment size >10 kb selected using a bluepippin system) according to the manufacturer’s instructions (Pacific Biosciences). Library quality was analysed by Qubit, and average fragment size was estimated using a Agilent 2,100 Bioanalyzer (Agilent, Santa Clara, CA, USA). SMRT sequencing was performed using a Pacific Biosciences RSII sequencer (PacBio, Menlo Park, CA) and standard protocols (MagBead Standard Seq v2 loading, 1×180 min movie) using P4-C2 chemistry.

### *De novo* genome assembly

Continuous long reads of >500 bp, with a quality value of >0.75, obtained from three SMRT sequencing runs were first merged into a single dataset. Next, the random errors in the long seed reads (seed length threshold 6 kb) were corrected by aligning the long reads against shorter reads from the same library using the hierarchical genome-assembly process (HGAP) pipeline^8^. The resulting corrected, preassembled reads were used for *de novo* assembly using Celera Assembler with an overlap-layout-consensus strategy^9^. Due to SMRT sequencing with very little variations in the quality throughout the reads^10^, hence no quality values were utilized during the assembly. the Quiver consensus algorithm^8^ was used to validate the quality of the assembly and to determine the final genome sequence. Finally, the ends of the assembled sequence were trimmed to circularize the genome. The completeness of the genomics data was assessed by BUSCO^11^.

### Gene prediction

The open reading frames (ORFs) were predicted using GeneMarkS^12^, which is a well-studied gene finding program used for prokaryotic genome annotation. Repetitive elements were identified by RepeatMasker^13^. Noncoding RNAs were predicted using rRNAmmer^14^ and tRNAs were identified using tRNAscan^15^.

### Genome annotations

Several complementary approaches were used to annotate the assembled sequences. The genes were annotated by aligning the sequence with sequences previously deposited in diverse protein databases including the National Center for Biotechnology Information (NCBI) non-redundant protein (Nr) database, UniProt/Swiss-Prot, Kyoto Encyclopedia of Genes and Genomes (KEGG), Gene Ontology (GO), Cluster of Orthologous Groups of proteins (COG), and protein families (Pfam). Additional annotation was carried out using the following databases: Pathogen Host Interactions (PHI), Virulence Factors of Pathogenic Bacteria (VFDB), Antibiotic Resistance Genes Database (ARDB), Carbohydrate-Active enZYmes (CAZy). Prophages were identified using the PHAge Search Tool (http://phast.wishartlab.com). Nr and GO annotation was carried out using Blast2GO, while protein families (Pfam) annotation was applied using Pfam_Scan (https://www.ebi.ac.uk/Tools/pfa/pfamscan/). An E-value of 1e^−5^ was used as the cut-off for all basic local alignment search tool.

### Code availability

Most of the custom codes used for dataset analysis were stated in the methods section, with default parameters used in other cases. Other softwares used in this study are as follows. The hierarchical genome-assembly process was performed using HGAP (smrtanalysis-2.3.0). The ORFs were predicted using Glimmer v3.02, and repetitive elements were identified by RepeatMasker (version open-4.0.5). Noncoding RNAs, such as rRNAs, were predicted using rRNAmmer (rnammer-1.2) and tRNAs were identified by tNRAscan (SE-1.3.1). More detailed information is provided in Table 1.

## Data Records

All of the raw reads for the Vqin-Q67 genome have been deposited in the NCBI Sequence Read Archive under accession number SRP108403 (Data Citation 1). All predicted genes and their functional annotations for the Vqin-Q67_ chromosome_1 and chromosome_2, have been depositied into GenBank under accession number GCA_002257545.1 (Data Citation 2).

## Technical Validation

**Figure 2:**
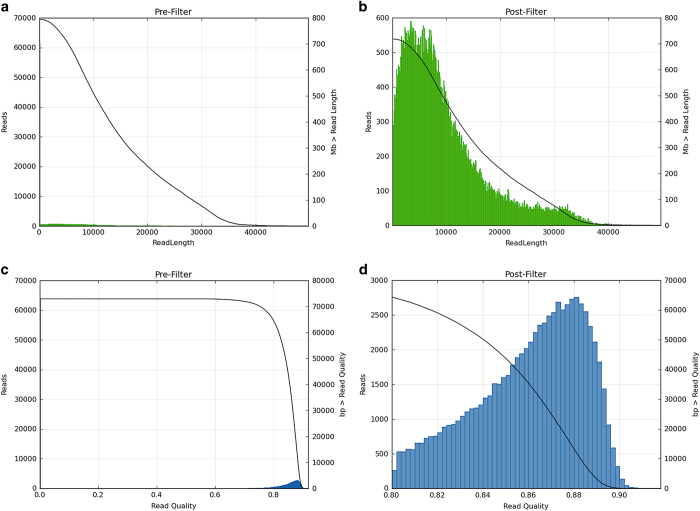
Quality control of the sequencing data. (**a**) Read length distribution before filtering. (**b**) Read length distribution after filtering. (**c**) Read quality distribution before filtering. (**d**) Read quality distribution after filtering.

**Figure 3:**
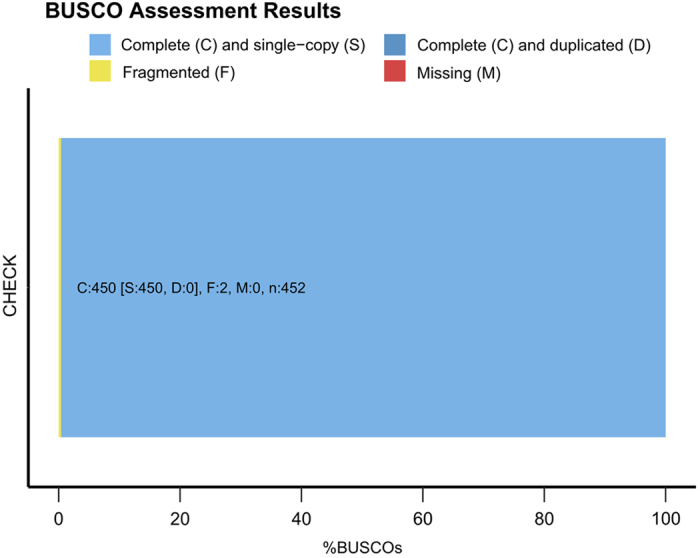
BUSCO assessment of the completeness of *Vibrio qinghaiensis sp.-Q67* gemome.

The presence of low quality or contaminated reads amongst the raw reads decrease the technical quality of the *de novo* assembly. To ensure the quality of the final assembly, raw reads and subreads were filtered to obtain clean reads for further assembly. Most of the short reads <100 bp were identified as adapter dimers (0–10 bp) or short fragment contamination (11–100 bp), filtering retained only raw reads with a length >100 bp, and an estimated accuracy of at least 80%. Although, this process may remove some true reads and reduce the number of reads from the raw pools, it resulted in a high-quality assembly with an average read length of 9,600 bp (Fig. 2b) and an accuracy of 0.859 (Fig. 2d). In the sub-read filtering step, we removed the adapter from the raw reads to obtain clean sub-reads with a mean length of 6,655 bp and a N50 of 8,487 bp (Supplementary Table 1). Results showed that the final assembly and annotation of Vqin-Q67 genome was 99.6% complete (Fig. 3), suggesting that most of the recovered genes could be classified as ‘complete and single-copy’.

**Figure 4:**
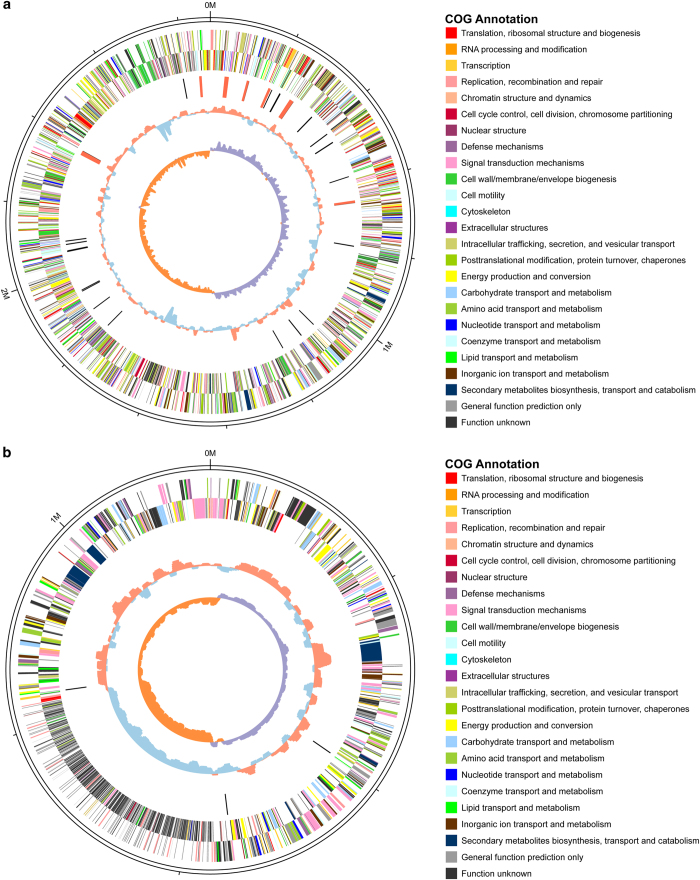
Circular representation of the *Vibrio qinghaiensis sp.-Q67* chromosome. (**a**) chromosome_1. (**b**) chromosome_2.

**Figure 5:**
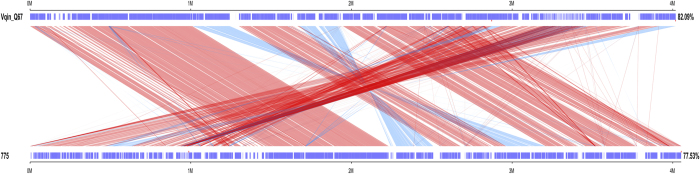
Synteny between *Vibrio qinghaiensis sp.-Q67* and *Vibrio anguillarum* 775. Nucleotide positions in the two genomes are indicated by 0, 1, 2, 3, and 4 M and the similarity regions are indicated by dark blue colour. Regions having undergone rearrangements are shown in gray. Red ribbons joining the two arcs indicate the synteny in the forward orientation, while the green ribbons indicate in the reverse orientation.

The *de novo* assembled genome is 4 Mb in size and is comprised of two chromosomes (chr_1 and chr_2) with an average GC content of 44.86 and 43.51%, respectively (Fig. 4). The two chromosomes were predicted to contain 2,591 and 1,372 protein-coding genes, respectively. All of the genes could be functionally annotated. The *lux* genes, which appear to be physically linked on chr_2, were identified as gene numbers 1,177 (*luxC*), 1,178 (*luxD*), 1,179 (*luxA*), 1,180 (*luxB*), 1,181 (*luxE*) and 1,182 (*luxG*). The *LuxCDABEG* arranement is typical of previously reported *lux* operons, including those in *Vibrio campbellii*^16^, *Vibrio cholerae*^17^ and *Photobacterium leiognathi*^18^. In addition, *luxR* (gene no. 1,329) also existed in the downstream of the *luxCDABEG* operon, which is consistent with other *Vibrio* species^5^. Based on 16S RNA gene sequence alignments (data not shown), Vqin-Q67 was found to be closely related to *Vibrio anguillarum* 775. *V. anguillarum* 775 is a highly virulent strain causing vibriosis in fish^19^. In contrast, Vqin-Q67, isolated from *Gymnocypris przewalskii* in Qinghai Lake, has a symbiotic relationship with its host^1^. Comparative genomic analysis and functional annotation supported the different natures of Vqin-Q67 and *V. anguillarum* 775. Synteny analysis showed frequent gene rearrangements between Vqin-Q67 and *V. anguillarum* 775, suggesting similarity regions of 82.09 and 77.53%, respectively (Fig. 5). This may account for their differences in pathogenicity to fish. Pathogen-host interaction proteins (PHIP) play an important role in modulating the host immune system^20^. However, whole-genome analysis of Vqin-Q67 in the current study showed that all proteins annotated as PHIP had only low identity (average 31%), which supports Vqin-Q67 being a symbiotic bacterium.

In summary, this dataset was optimized using the above parameters and quality control measures, and therefore, should be free from errors. Furthermore, the comparative genomic and annotation analyses performed using this dataset provides powerful evidence for its high level of accuracy and practicability.

## Additional information

**How to cite this article:** Gong, L. *et al.* Complete genome sequencing of the luminescent bacterium, *Vibrio qinghaiensis sp. Q67* using PacBio technology. *Sci. Data* 5:170205 doi:10.1038/sdata.2017.205 (2018).

**Publisher’s note:** Springer Nature remains neutral with regard to jurisdictional claims in published maps and institutional affiliations.

## Supplementary Material



Supplementary Table 1

## Figures and Tables

**Table 1:** Versions and parameters of the software used in this study.

**Dataset/ Software**	**Function**	**version**
Nr	Protein annotation	2017.03.09
KEGG	Functional annotation	Release 76.0
GO	Functional annotation	2016 march
COG	Bacterial homologous protein annotation	2015.07.24
KOG	Fungal homologous protein annotation	2015.07.24
PHI	Host pathogen interaction	version 4.0
VFDB	Prediction of virulence factors	2015
ARDB	Resistance gene prediction	ardbAnno1.0
antismash	Prediction of secondary metabolic gene cluster	antismash-2.1.1
HGAP	Sequence assembly	smrtanalysis-2.3.0
rRNAmmer	rRNA Prediction	rnammer-1.2
tRNAscan	tRNA Prediction	tRNAscan-SE-1.3.1
PHAST	Prophage prediction	phage_finder_v2.1
signalP	Secretory protein prediction	signalp-4.1
TransposonPSI	Transposon prediction	20100822
IslandViewer4	Gene Island analysis	IslandViewer 4
Blast	Sequence comparison	2.6.0
Circos	Genome map	v0.62
Perl+SVG	Genome synteny	
