# Supplementary material for: Complete genome sequencing of the luminescent bacterium, Vibrio qinghaiensis sp. Q67 using PacBio technology
Source: Sci Data. 2018 Jan 16;5:170205. doi: 10.1038/sdata.2017.205 (PMC5769541; doi:10.1038/sdata.2017.205)
Supplement: Supplementary Table 1 [file sdata2017205-s2.docx]

**Supplementary Table 1.** Summary of the subreads information in genome of *V. qinghaiensis sp.-Q67.*

| **Sample** | **Reads Bases** | **Number of Reads** | **Mean length** | **Reads N50** |
| --- | --- | --- | --- | --- |
| **Vqin_Q67** | 719272306 | 108079 | 6655 | 8487 |
